# Minimizing thermal damage to vascular nerves while drilling of calcified plaque

**DOI:** 10.1186/s13104-019-4381-2

**Published:** 2019-06-14

**Authors:** Seifollah Gholampour, Keyvan Hajirayat

**Affiliations:** Department of Biomedical Engineering, Islamic Azad University-Tehran North Branch, Tehran, Iran

**Keywords:** High speed drilling, Vascular nerve, Finite element method, Calcified plaque, Thermal stroke

## Abstract

**Objective:**

Drilling of calcified plaque (DCP) inside the artery is a method for removing calcified plaques. This study investigated the effect of drill. To validate the maximum temperature calculated by computer simulation, this value was also measured by an experimental on a phantom model.

**Results:**

Increasing drill bit diameter during drilling would increase the temperature in vascular nerves. In a drill bit with a diameter of 4 mm, the risk of thermal necrosis in vascular nerves of the artery wall decreased by 8.57% by changing the drill from WC to NT. The same value for a drill bit with a diameter of 6 mm was 10.17%. However, the trend of the generated temperature in the vascular nerves did not change significantly with change of the material and diameter of the drill bit. The results showed that for DCP with the least risk of thermal necrosis in vascular nerves and subsequently the lowest risk of restenosis, coagulation and thermal stroke of the patient, the best option is to use a drill bit with a diameter of 4 mm and NT material for drilling.

## Introduction

**Fig. 1:**
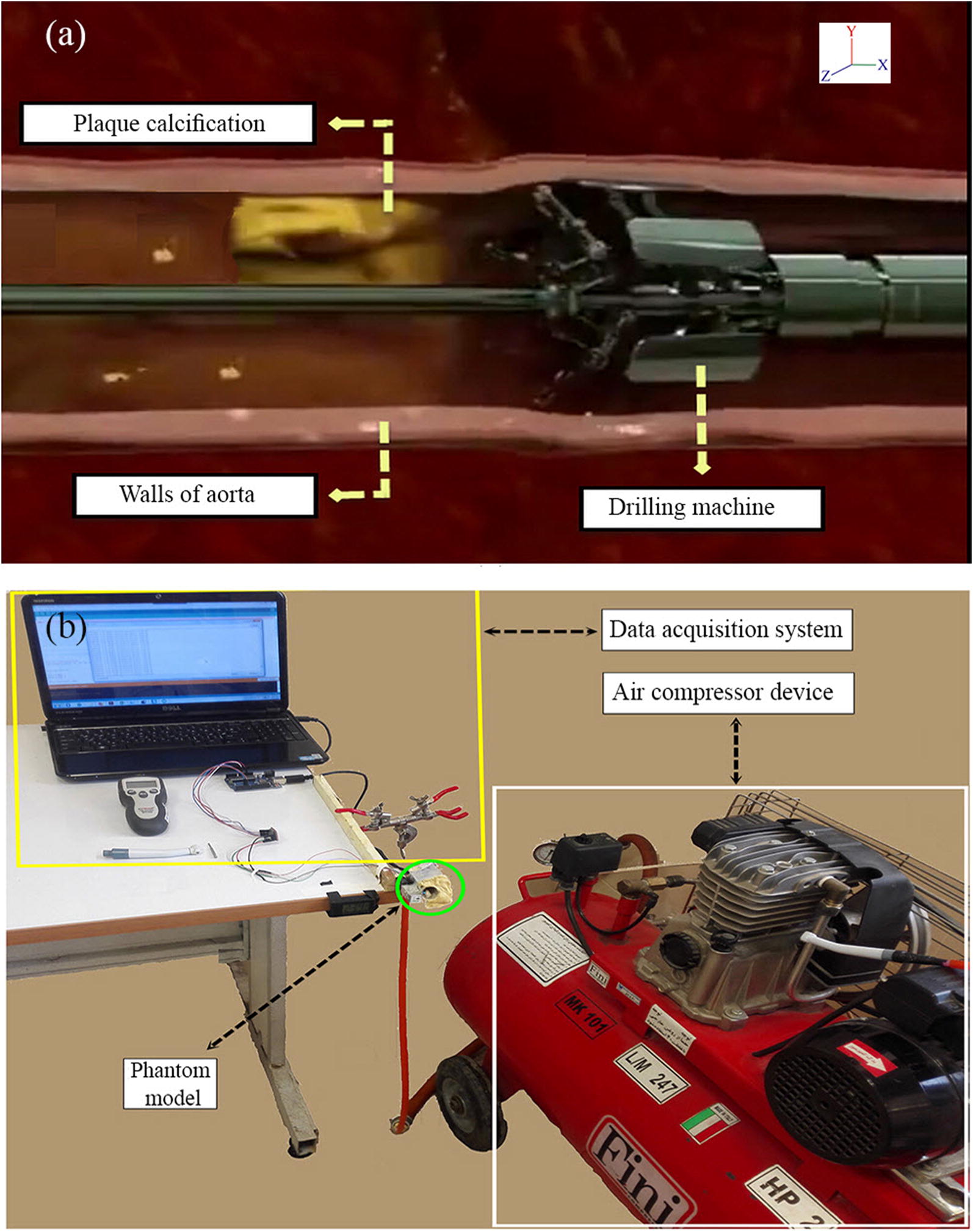
**a** The process of high speed DCP, **b** the equipment used to perform the high speed DCP in the artery

The vascular nerves consisted of small neural fibers, which were responsible for the contraction of smooth muscle in the wall of the arteries [1]. These nerve cells are responsible for controlling vasoconstriction processes and vasodilation [2]. The accumulation of calcified plaque on the wall of the arteries, especially the aorta, can obscure the regulation of these two processes, leading to blockage in the lumen and stroke of the patient. One of the new methods suggested in recent studies for treating these patients is high-speed drilling in the artery to eliminate the blockage of calcified plaques [3–5] (Fig. 1a).

There have been many studies of the interactions between biological fluids and the walls of arteries or their interfaces in the body [6–9]. Some studies have focused on optimal parameters in the process of drilling in the body [10–12]. However, very limited studies have specifically sought to achieve optimal parameters during drilling of calcified plaque (DCP) in arteries. Lin et al. [13] examined the effective factors in the incidence of drill bit stuck during DCP. Nakao et al. [14] designed a device for grinding a stiffened egg shell without damaging its whites, which was actually simulated by the DCP process. Abdel-Wahab et al. [15] examined the effect of DCP on drug stents. Ramazani-Rend et al. [16] assessed the effect of cavitation during the DCP process. Some researchers evaluated the effect of rotational speed during the drilling on restenosis after DCP [4, 17]. Lovik et al. and Abaraham et al. numerically and experimentally examined the damage of plaque drilling in development of arterial thermal damage in orbital atherectomy [18, 19]. Helgeason et al. investigated particle trajectory and fluid flow in orbital atherectomy [20].

One of the main considerations that should be considered when using DCP is to control and prevent temperature rise in the artery wall during the drilling process. Because the lack of control over the heat generated by drilling leads to thermal necrosis of these vascular nerves in the wall of the arteries, it provides post-treatment restenosis and coagulation and permanent damage to the artery walls [21–23]. In this study, the effect of material and diameter of the drill bits during DCP was studied to achieve minimum heat generated in the vascular nerves.

## Main text

### Methods

To investigate the effect of the drill bit in the DCP process, two drill bits with tungsten carbide (WC) and nitride-titanium (NT) materials, the most common drill bit materials for biocompatibility, were used. To evaluate the effect of drill bit diameter in each material, two drill bits with a diameter of 4 and 6 mm were used. The reason for using these two diameters for a drill bit in this study was that in previous studies for drilling inside the artery, according to the common internal diameter of the arteries that were blocking, diameters were proposed in this range [4]. To obtain the temperature of the artery wall during drilling, computer simulation was used to determine the damage to the vascular nerves. According to previous studies, one of the most suitable computer simulations is in the finite element method (FEM) [24–30].

#### Computer simulation

**Fig. 2:**
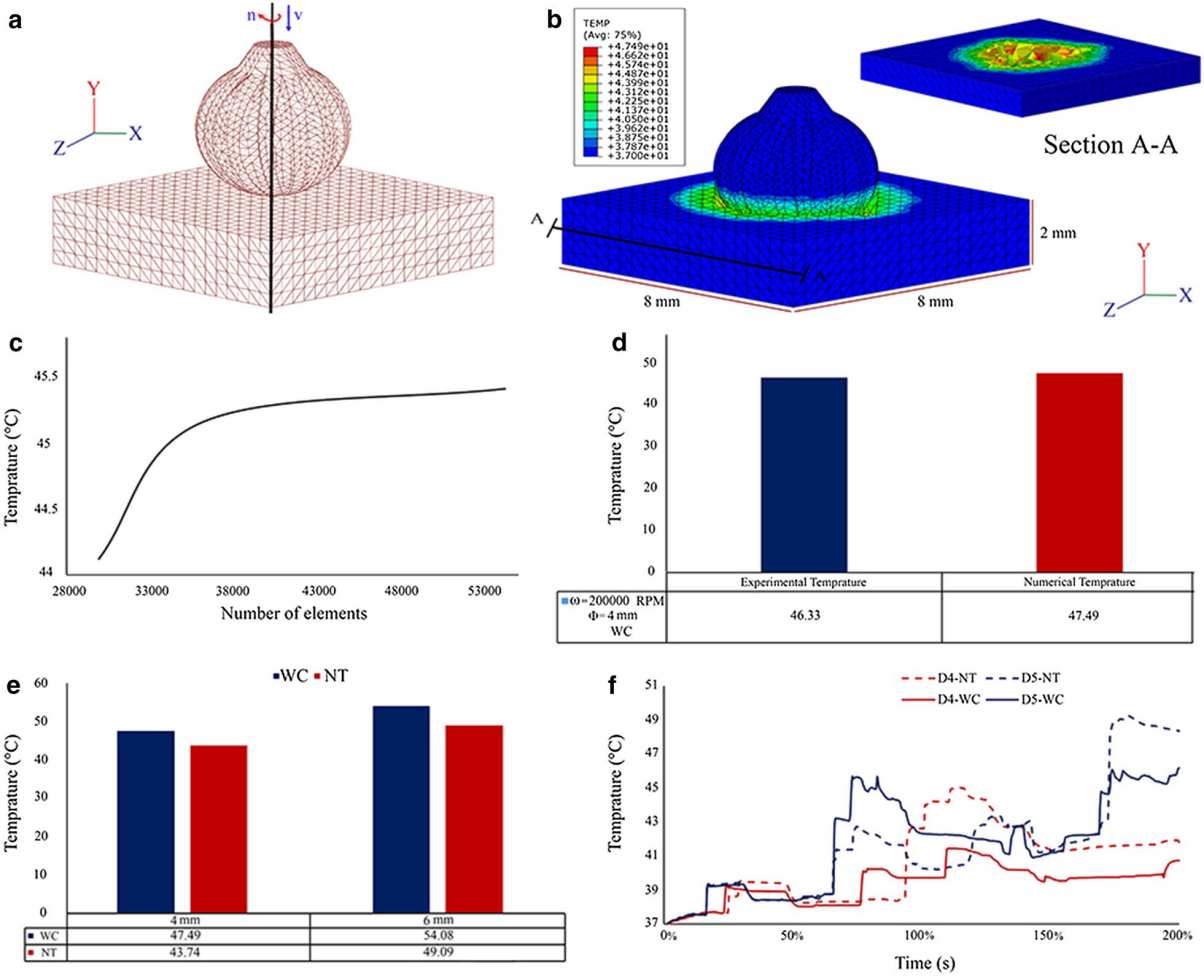
**a** Mesh and boundary condition applied to the models, **b** distribution of temperature with drill bit of 4 mm diameter and WC material and cross section of bone after high speed drilling, **c** grid independence and convergence, temperature results of computer simulation based on number of elements, **d** compare of temperature results-computer simulation (red color) and experimental (blue color) for drill bit with 4 mm dimeter and WC material, **e** temperature results of DCP with four drill bits with 4 and 6 mm diameters and with WC and NT materials, **f** rise of temperature on the vessel walls induced by four drill bits

FEM method is a one of the most common methods to simulation in biological areas and especially in drilling field [31, 32]. This numerical simulation uses mesh production methods to divide a complex problem into small elements and mostly uses for solid terms analysis. In the past, a lot of experiments were needed to achieve the results of drilling, however, in FEM simulation can be obtained with higher precision [33–35]. Therefore, a Lagrangian finite element model of DCP was developed in ABAQUS/Explicit software. To control the element deletion a dynamic failure criterion should be applied. In this research for thermal simulation of the DCP process, a 3D model of dynamic temp-disp explicit was placed under a numerical FEM analysis. To do this, the calcified plaque was modeled as a rectangular cube with dimensions of 8 mm in length, 2 mm in width and 8 mm in height with SOLIDWORKS 2018 software. Then the drill bits were in 3D modeling mode. Next, two drill bits with a diameter of 4 mm and materials of WC and NT were modeled and then the same drill with a diameter of 6 mm and materials of WC and NT modeling 3D. Material characteristics of drill bits and bone are presented in Table 1 [36, 37]. In each of four modes, drill bit models, calcified plaque and artery for meshing and FEM analysis were transferred to ABAQUS software Version 6.14 (Fig. 2a).

**Table 1:** Mechanical and thermal properties of workpiece and drill bits

Material	Plaque	Drill bit (WC)	Drill bit (NT)
Density (kg/m^3^)	2050	1800	4500
Young modulus (GPa)	13.80	540	450
Passion’s ratio	0.33	0.28	0.25
Thermal conductivity (W/m °C)	0.51	40	19
Specific heat (J/kg °C)	516	203	603
Expansion (1/°C)	2.7e−6	4.7e−6	9.4e−6
Initial yield strength	50	–	–
Hardening coefficient	101	–	–
Coefficient of strain rate	0.03	–	–
Coefficient of strain hardening	0.08	–	–
Coefficient of thermal softening	0.9	–	–

The contact behavior between the drill bit and the workpiece was considered as surface to surface and was considered a penalty method. Also, the coulomb friction coefficient between the drill bit and the workpiece was also considered to be 0.3 [4]. Also, on the contact surface between the drill bit and calcified plaque is water with convection heat transfer coefficient of 103.20 W/m^2^ °C. The Johnson-Cock material definition was used to define how the model was damaged during the drilling process. The values of the Johnson-Cock model for bovine cortical femur bovine are shown in Table 1 [36]. To penetrate the drill bit into the workpiece, its linear motion along the axes X and Z, as well as the rotational motion around the X axis and Z, were constrained (Fig. 2a). Also, all four vertical surfaces of the workpiece model were considered in all linear directions around all the fix axes. According to previous research, DCP process should be done in high speed mode for best results [13, 14]. Therefore, rotational speed of 200,000 RPM, which is one of the most recommended high speed drilling values in previous studies, was used in this study [14].

After performing the computer analysis process, to ensure the accuracy of the simulation, grid independence, and convergence of the results were examined. Figure 2c, shows that, as the number of elements increases, the numerical difference between the calculated values for the temperature decreases strongly. The low difference between the medium and fine meshes indicates that the conditions for the convergence of responses and the independence of the network are used in computer simulation, is acceptable.

#### Experimental procedure

To validate the results of computer simulation, the maximum temperature during the DCP process was measured on a phantom model experimentally. An overview of the DCP process is shown in Fig. 1a. The experimental procedure included a measurement system, a high speed drilling system, and a phantom model (artery and calcified plaque).

To record the maximum experimental temperature, a K-type thermocouple was used with and to record the rotational speed of the drill bit at instances, the non-contact tachometer (MTC-642, Marmonix Inc. Canada) was used. Each test was repeated three times to ensure no errors were observed in the measured data. For high speed drilling, a dental turbine machine (Being.inc, China) with a rotational speed of 120,000 to 320,000 RPM was used, the power supply was an air compressor (Fig. 1b). Dental turbines with compressed air pressure (0.5–2.5 bar) are able to rotate a drill bit under 120,000 to 400,000 RPM. This hand piece was used to drilling or grinding machining teeth. Therefore, at the present research, dental turbine was used to provide the desired speed and power. To simulate the DCP process, a phantom model consisting of an artery and a calcified plaque was made. For the artery model, a PVC tube with 30 mm diameter and with similar physical characteristics of artery (Young’s modulus = 45 kPa) was used. For calcified plaque model, a cube bone of bovine with size of 8 × 8 × 2 mm was implanted on the wall of PVC tube (Fig. 2a, b). Inside the PVC tube, water flowed to simulate the actual condition of the blood in the arteries during the test.

The calcified plaque was mounted on the tube wall. Then, the load cell was attached to the tube wall to measure radial force (Y axis). To simulate the actual condition of the blood, the water flow with 37 °C temperature and 12 mmHg pressure had flowed into the tube. Then drill bits with high rotational speeds (120–200 K-rpm) and with constant feed rate in X axis were penetrated linearly. During DCP process, the thermocouple and the load cell were recorded the temperature and radial force, respectively.

### Results and discussion

For validating the results of the computer simulation, the maximum temperature data measured by the thermocouple in the experimental method for a 4 mm diameter WC drill was compared with the similar results that were calculated by FEM simulation of the same model. According to Fig. 2d, since the difference of the results of simulation is less than 2.5% compared to the experimental data for the intended drill, the assumptions used during the computer simulation process and acceptable. The diagrams and trend patterns of temperature–time and temperature distribution at the artery walls are only available in computer simulation and cannot be measured in the experimental methods.

In the WC material, the maximum temperature in the artery walls for drill bits with diameters 4 and 6 is 47.49 °C and 54.08 °C, respectively (Fig. 2e). Similar temperatures in the NT material were also 43.74 °C and 49.09 °C, respectively. The results of Fig. 2e, a show that during DCP, with increasing drill bit diameter from 4 to 6 mm, temperature in vascular nerves of artery walls increased by 12.23% and 13.88% for NT and WC materials, respectively. Therefore, during the DCP process for each drill material, by changing the diameter of the drill bit, temperature has changed significantly in the vascular nerves of the artery walls.

According to Fig. 2b and e, in DCP with drills of either diameter, with change in material, temperature has changed significantly in the vascular nerves of the artery walls. During the DCP with a 4 mm diameter drill, when the material changed from WC to NT, the temperature in the vascular nerves was reduced by 8.57%. The same number for a DCP with a drill with a diameter of 6 mm was 10.17%.

Previous studies have shown that an increase in temperature above 43 °C leads to a change in the blood coagulation process, which will cause a block of the artery and a patient’s thermal stroke [3]. In this study, the fluid flow temperature was also measured during drilling, and the results confirmed that blood temperature during the drilling operation did not rise above 37 °C. On the other hand, previous studies have shown that the risk of thermal necrosis is one of the most important concerns for damage to the artery wall vascular nerves [5] because of the thermal damage to the artery wall vascular nerves, the field of occurrence of restenosis, coagulation and blockage may occur [3, 5]. Therefore, the main focus of this study was to calculate the temperature values in the artery wall during the DCP process.

The results of Fig. 2e, showed that during DCP, with increasing diameter of the drill bit from 4 to 6 mm, the heat generated in the vascular nerves of the artery wall, and subsequently the risk of thermal damage in these vascular nerves increased for the drill bit with WC material by 13.9%. The same number for a drill bit with NT material was 12.2%. This means that by increasing the diameter of the drill bit from 4 to 6 mm for a DCP with a drill bit with WC, the risk of thermal necrosis in the vascular arteries of the vascular nerves is about 1.14 times higher than the NT drill bit.

The results of Fig. 2e, showed that during the DCP process with a drill bit with a diameter of 4 mm, with a change in drill bit material from WC to NT, the temperature in the vascular nerves decreased by 8.57%, and the same value for the drill bit with a diameter of 6 mm was 10.17%. The difference in temperature in vascular nerves in two different materials of the drill bit is due to the difference in the heat transfer coefficient of the drill bit. Table 1 shows that the heat transfer coefficient of a drill bit in WC and NT materials is 40 and 19 W/m K, respectively. The results of Fig. 2e, showed that using a drill bit with NT with a fixed diameter, could generate less than 1.60 times heat than WC. Consequently, the likelihood of the occurrence of thermal necrosis in vascular nerves and subsequent complications such as restenosis, coagulation and, ultimately, the patient’s thermal stroke, is 1.60 times less when using a drill bit with NT material than a drill bit with a WC material.

Figure 2f, shows temperature–time changes in two cycles of the DCP process. The results showed that all the graphs demonstrate an upward trend in temperature as the drilling time increases. First, with increasing drilling time, the temperature of the vascular nerves of the artery wall increases until it hits its maximum value, and then, falls until the end of the drilling process. The results showed that the diameter and material variation of the drill bit had no significant effect on the temperature–time trend.

### Conclusion

The results showed that the diameter and material of the drill bit during the DCP process are both effective factors in thermal damage in the vascular nerves. The results also showed that using a larger diameter drill bit and materials with a higher heat transfer coefficient would increase the amount of heat generated in the artery wall. But, given that drilling time is one of the most important factors in increasing temperature, it is necessary that in a shorter time, more volume of calcified plaque is eliminated. Therefore, the results of this study showed that to keep the drilling time in just 15 s, it is better to use a drill bit with a diameter of 4 mm and of NT material.

## Limitations

We did not consider the effects of the drill bit angle and geometry on the thermal necrosis of the artery wall vascular nerves during the DCP process. In previous studies, thermal stress has been investigated in a rotational atherectomy process, however, it is suggested that future studies should investigate mechanical impact on the blood (hemolysis) during this process [38].

## Data Availability

All data used for the present study are available and could be requested from the authors.

## Abbreviations

DCP: drilling of calcified plaque
WC: tungsten carbide
NT: nitride-titanium
FEM: finite element method
